# Telestroke systems and thrombolysis decisions: diagnostic uncertainty, treatment thresholds, and stroke mimics

**DOI:** 10.3389/fneur.2026.1889300

**Published:** 2026-09-15

**Authors:** Wenjing Zhang, Likun Wang, Siying Ren, Zhao Yang, Guofeng Wu, Ke Gao

**Affiliations:** 1 Emergency Department, The Affiliated Hospital of Guizhou Medical University, Guiyang, Guizhou, China; 2 Key Lab of Acute Brain Injury and Function Repair, Guizhou Medical University, Guiyang, Guizhou, China; 3 Department of Neurology, The Affiliated Yongchuan Hospital of Chongqing Medical University, Chongqing, China; 4 Department of Critical Care Medicine, The Affiliated Liupanshui Hospital of Guizhou Medical University, Liupanshui, Guizhou, China

**Keywords:** acute ischemic stroke, intravenous thrombolysis, stroke mimics, telemedicine decision-making, telestroke

## Abstract

**Background:**

Telestroke expands access to specialist stroke care and is associated with increased use of intravenous thrombolysis (IVT). Whether this reflects improved access alone or also changes how clinicians act under diagnostic uncertainty remains unclear.

**Purpose:**

To review evidence on telestroke-associated thrombolysis, stroke mimics, and decision-making, and to examine the hypothesis that telestroke may influence the practical threshold for IVT.

**Methods:**

We conducted a narrative review of studies addressing telestroke implementation, IVT utilization, stroke mimic frequency and safety, remote neurological assessment, and decision-related features of telestroke care. Evidence was synthesized around three proposed domains: risk asymmetry, information constraints and diagnostic friction, and distributed responsibility with workflow pressure.

**Results:**

Telestroke is consistently associated with greater thrombolysis utilization, while stroke mimics remain common among both evaluated and treated patients. Serious hemorrhagic complications after thrombolysis of stroke mimics are uncommon. However, existing studies are predominantly observational and do not directly measure clinicians’ diagnostic confidence or treatment thresholds. Higher IVT rates and mimic treatment may therefore reflect improved specialist access, faster workflows, case mix, or other system-level factors rather than a lower threshold for treatment.

**Conclusion:**

Current evidence does not demonstrate that telestroke lowers the thrombolysis threshold. Instead, a treatment-threshold shift should be regarded as a testable hypothesis. Future studies should directly measure diagnostic confidence, estimated stroke probability, competing diagnoses, and treatment decisions to determine how telestroke influences clinical decision-making under uncertainty.

## 1 Introduction

Acute ischemic stroke remains a major cause of mortality and long-term neurological disability worldwide, and the continuing global burden of stroke underscores the importance of improving access to effective acute treatment (1). For patients with eligible acute ischemic stroke, intravenous thrombolysis remains an established reperfusion strategy, and its clinical benefit is strongly time dependent; shorter treatment delays are consistently associated with better neurological outcomes and reduced disability (2, 3). Consequently, rapid access to stroke expertise has become a central component of modern acute stroke systems. Nevertheless, substantial disparities in specialist availability persist, particularly in community and rural hospitals where on-site vascular neurologists may not be continuously available. Telestroke was developed to address this gap by enabling remote stroke specialists to participate in real-time neurological assessment, neuroimaging review, and treatment decision-making at hospitals without dedicated stroke expertise (4, 5).

The expansion of telestroke has substantially altered the delivery of reperfusion therapy. Registry studies, implementation analyses, and network-based evaluations have repeatedly shown that access to telestroke is associated with greater use of intravenous thrombolysis, while some systems have also reported reductions in unnecessary interhospital transfer and improved treatment access at the presenting hospital (6–9). These observations represent an important achievement in stroke service delivery and demonstrate the capacity of telemedicine to overcome geographic and organizational barriers to specialist care. Telestroke programs may facilitate treatment through several mechanisms, including earlier neurological consultation, more rapid interpretation of imaging, improved implementation of stroke protocols, and greater confidence among local clinicians when determining eligibility for reperfusion therapy (4, 10, 11). However, higher thrombolysis utilization does not, by itself, establish why more patients are treated. Increased treatment rates may reflect improved access to expertise, more complete identification of eligible patients, more efficient workflows, changes in referral patterns, or greater adherence to guideline-based stroke pathways. They therefore should not automatically be interpreted as evidence that clinicians are applying a lower treatment threshold.

This distinction becomes particularly important because hyperacute stroke treatment frequently proceeds before diagnostic certainty is complete. A variety of non-vascular conditions—including epileptic seizures, migraine with aura, functional neurological disorders, toxic or metabolic disturbances, and vestibular syndromes—can produce acute focal neurological symptoms that closely resemble ischemic stroke (12, 13). These conditions, collectively referred to as stroke mimics, constitute a substantial proportion of patients initially assessed for suspected stroke and are also encountered frequently within telestroke pathways (14, 15). Importantly, some patients who are ultimately diagnosed with stroke mimics receive intravenous thrombolysis before the final diagnosis becomes apparent. Available cohort and meta-analytic evidence generally indicates that symptomatic intracranial hemorrhage and other severe complications occur less frequently in thrombolysed stroke mimics than in patients with confirmed ischemic stroke (16–18). This creates an asymmetric clinical decision problem: withholding or delaying reperfusion in a true ischemic stroke may result in irreversible neurological injury, whereas thrombolysis in a patient ultimately diagnosed with a stroke mimic appears to carry a comparatively lower probability of catastrophic hemorrhagic harm (2, 17).

Telestroke may be particularly relevant to how this uncertainty is managed. Remote stroke assessment is performed under substantial time pressure and may rely on video-mediated examination, variable collateral history, bedside facilitation by local clinicians, and rapid interpretation of imaging. Although remote neurological evaluation is generally feasible and can achieve good overall diagnostic performance, certain elements of the examination and clinical context may be more difficult to capture than during direct bedside assessment (4, 19, 20). In addition, treatment decisions are often distributed between the remote stroke specialist and the local emergency team, creating a decision environment that differs from conventional face-to-face assessment. These features could plausibly influence how clinicians weigh uncertainty, treatment delay, and competing diagnostic errors. Nevertheless, the available evidence is predominantly observational, and existing telestroke studies have generally measured treatment rates, workflow metrics, outcomes, or final diagnoses rather than the probability threshold at which individual clinicians decide to administer thrombolysis (7, 8, 14). Accordingly, neither an increased rate of thrombolysis nor a greater proportion of treated stroke mimics can independently demonstrate that clinicians are accepting a lower probability of true ischemic stroke before initiating treatment.

The central question of this review is therefore not whether telestroke has been proven to lower the thrombolysis threshold, but whether the available evidence is compatible with a possible shift in the practical treatment threshold and whether this hypothesis warrants direct investigation. We treat threshold shift as a conceptual and testable hypothesis rather than an established consequence of telestroke. Specifically, we synthesize evidence regarding thrombolysis utilization in telestroke systems, the frequency and treatment of stroke mimics, the safety profile of thrombolysis under diagnostic uncertainty, and the characteristics of remote clinical decision-making that may influence treatment behavior. We also distinguish observed associations from proposed behavioral mechanisms, consider alternative explanations for higher treatment rates, and identify methodological approaches that could directly measure treatment thresholds in future studies. By doing so, this review seeks to move the discussion beyond whether telestroke simply increases access to thrombolysis toward a more precise question: how does the remote care environment influence treatment decisions when diagnostic certainty remains incomplete?

The overall conceptual framework of this review is summarized in the Graphical Abstract (Figure 1).

**Figure 1:**
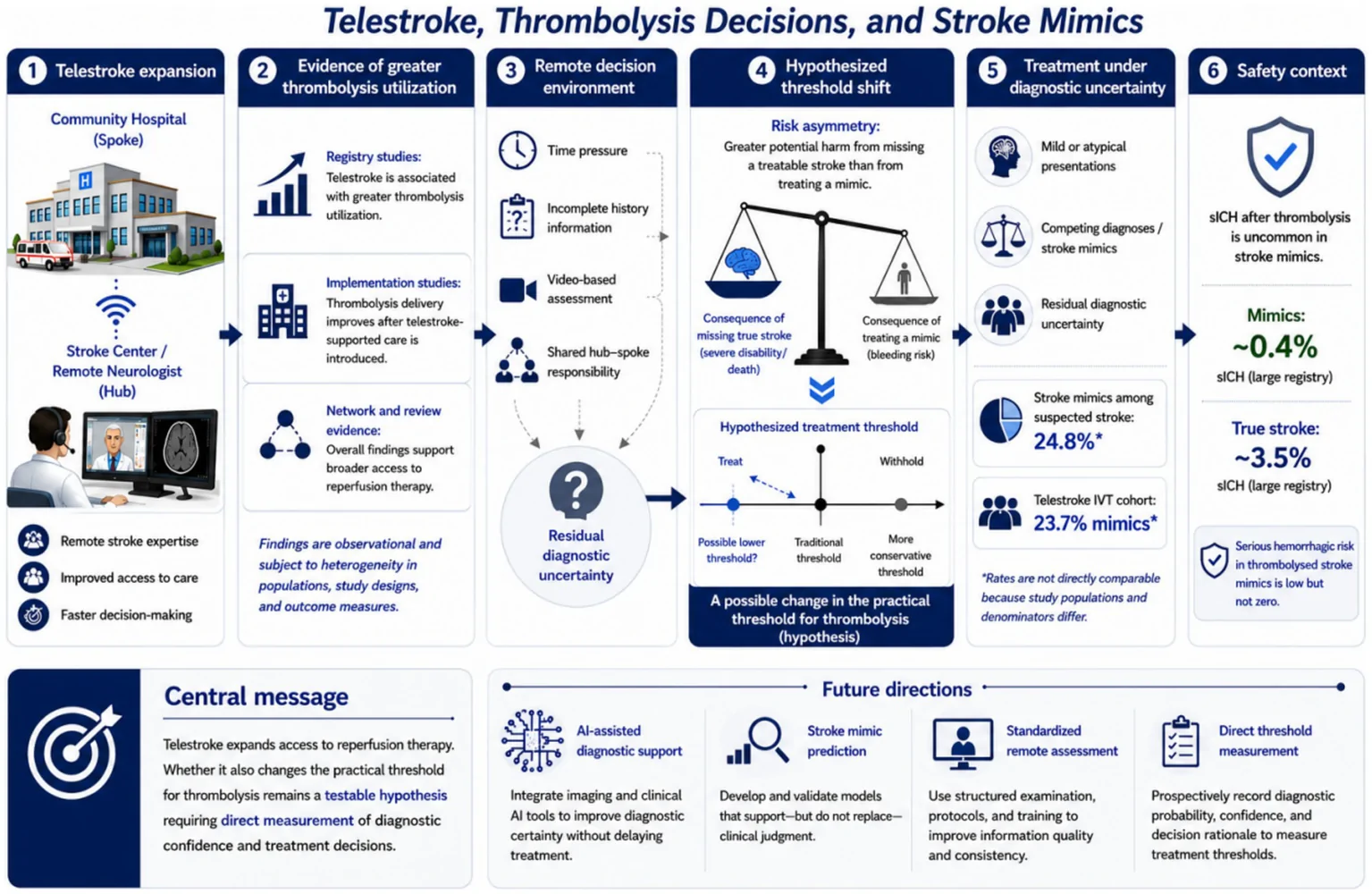
Conceptual overview of telestroke, thrombolysis decision-making, and stroke mimics.

Telestroke expands access to specialist stroke care and is associated with greater use of intravenous thrombolysis (IVT). Remote treatment decisions are made within a time-sensitive environment characterized by incomplete information, video-based assessment, and shared hub–spoke responsibility. These features, together with the asymmetric consequences of missing a treatable ischemic stroke versus treating a stroke mimic, provide the conceptual basis for a possible shift in the practical treatment threshold. Stroke mimics remain relevant to this decision context, although serious hemorrhagic complications after thrombolysis of mimics are uncommon. The proposed threshold shift is presented as a testable hypothesis rather than an established effect. Quantitative values shown in the graphical abstract correspond to findings reported and cited in the main text. Future priorities include AI-assisted diagnostic support, stroke mimic prediction, standardized remote assessment, and direct measurement of treatment thresholds.

## 2 Methods

### 2.1 Review design and scope

This study was conducted as a narrative review aimed at integrating clinical and conceptual evidence regarding telestroke, intravenous thrombolysis, stroke mimics, and treatment decision-making under diagnostic uncertainty. The review was not designed as a systematic review or meta-analysis; therefore, no protocol registration, formal risk-of-bias assessment, or quantitative pooling of effect estimates was undertaken. Instead, the literature was synthesized to identify consistent clinical observations, areas of uncertainty, and plausible mechanisms through which the telestroke environment may influence thrombolysis decisions. The overall approach was guided by established principles for the conduct and reporting of narrative reviews (21).

The scope of the review was intentionally restricted to literature relevant to four interconnected domains: (1) the association between telestroke implementation and thrombolysis utilization; (2) the frequency, characteristics, and treatment of stroke mimics in acute stroke pathways; (3) the safety of thrombolysis when administered to patients subsequently diagnosed with stroke mimics; and (4) characteristics of remote clinical assessment that may influence decision-making when diagnostic certainty is incomplete.

### 2.2 Literature search strategy

Relevant literature was identified through targeted searches of PubMed up to August 5, 2026. Search terms were used individually and in combination and included telestroke, telemedicine, acute ischemic stroke, intravenous thrombolysis, alteplase, tenecteplase, stroke mimic, diagnostic uncertainty, remote neurological examination, NIHSS, clinical decision-making, and treatment threshold. Reference lists of relevant reviews, guidelines, and key primary studies were additionally examined to identify potentially informative publications not captured during the initial searches.

Particular attention was given to studies directly evaluating telestroke-supported acute stroke care, including registry analyses, prospective or retrospective cohort studies, before-and-after implementation studies, multicenter studies, and meta-analyses. Studies examining stroke mimics outside telestroke settings were also considered when they provided relevant information regarding mimic epidemiology, differential diagnosis, or the safety of intravenous thrombolysis. Scientific statements and clinical guidance addressing telestroke organization, remote neurological assessment, and acute reperfusion pathways were used to provide contextual information regarding current clinical practice (4).

### 2.3 Evidence selection and prioritization

Evidence was selected according to its relevance to the specific questions addressed in this review rather than through a formal systematic eligibility framework. Priority was given to studies that reported at least one of the following outcomes: thrombolysis utilization in telestroke systems; reperfusion treatment before and after telestroke implementation; frequency of stroke mimics among telestroke consultations or thrombolysis-treated patients; hemorrhagic complications among thrombolysed stroke mimics; diagnostic accuracy of remote stroke assessment; or factors potentially affecting treatment decisions in telemedicine-supported stroke care.

Where multiple studies addressed the same issue, greater weight was given to recent multicenter cohorts, large registries, meta-analyses, and studies with clearly described diagnostic adjudication. Telestroke-specific evidence was prioritized over general acute stroke literature when the question concerned remote clinical practice. Conversely, broader stroke literature was included when telestroke-specific evidence was limited, particularly for the safety of thrombolysis in stroke mimics and the time-dependent consequences of delayed reperfusion.

Because studies differed substantially in population selection, definitions of stroke mimic, telestroke configuration, thrombolytic agent, and outcome ascertainment, numerical estimates were interpreted in their original study contexts rather than combined into a new pooled estimate. Differences between studies were considered part of the evidence landscape and were used to identify areas requiring further prospective investigation.

### 2.4 Narrative synthesis and development of the behavioral framework

The proposed behavioral framework was developed through an iterative narrative synthesis of recurrent decision-related themes identified across the telestroke, stroke mimic, thrombolysis, and remote-assessment literature. Importantly, the behavioral domains presented in this review were not derived from a previously validated classification system and should not be interpreted as an established taxonomy of telestroke behavior. Rather, they were used as an organizing framework to integrate recurring features of clinical decision-making that could plausibly influence treatment choices under uncertainty.

Three principal domains were retained because they were recurrent, conceptually distinct, and directly relevant to telestroke practice. Risk asymmetry was defined as the unequal perceived consequences of withholding thrombolysis from a patient with true ischemic stroke compared with administering thrombolysis to a patient with a stroke mimic. Information constraints and diagnostic friction referred to limitations in the availability, completeness, or reliability of clinical information during remote assessment, including video-mediated examination, incomplete collateral history, and difficulties assessing subtle neurological findings. Distributed responsibility and workflow pressure referred to the sharing of diagnostic and therapeutic decisions between hub and spoke teams and to the influence of time-sensitive, protocol-driven workflows on treatment decisions.

These domains were used to interpret, rather than establish causation from, the observational findings identified in the literature. In particular, increased thrombolysis utilization or a higher frequency of treated stroke mimics was not considered direct evidence of a lower treatment threshold. Instead, such observations were evaluated as indirect evidence relevant to a threshold-shift hypothesis, which was examined alongside alternative explanations such as improved specialist access, better identification of eligible patients, workflow optimization, changes in referral patterns, and greater adherence to reperfusion protocols. The resulting framework was therefore intended to generate testable hypotheses for future research rather than to imply that behavioral threshold changes have already been demonstrated.

## 3 Results

### 3.1 Telestroke and thrombolysis utilization

Across multiple healthcare settings, the implementation of telestroke has been consistently associated with greater use of intravenous thrombolysis and broader access to reperfusion therapy. This pattern has been observed in registry analyses, health-system implementation studies, claims-based evaluations, and meta-analytic evidence, although the magnitude of the association varies according to network structure, patient population, and study design (6–9).

One of the most recent registry-based analyses was reported by Stamm et al., who evaluated patients with acute ischemic stroke across hospitals participating in a statewide stroke registry. Telestroke involvement was associated with a higher likelihood of receiving intravenous thrombolysis, with an adjusted odds ratio of 1.61 (95% CI, 1.17–2.23) compared with care without telestroke activation (7). Importantly, this study was observational and therefore demonstrates an association between telestroke involvement and treatment utilization rather than a causal effect of remote consultation on clinicians’ treatment thresholds.

Similar findings have been reported in broader comparisons of hospitals with and without telestroke capability. Wilcock et al. examined reperfusion treatment patterns across a large US hospital sample and found that hospitals with telestroke capacity were more likely to provide reperfusion therapy than hospitals without such capacity (8). These findings support the interpretation that telestroke can reduce structural barriers to time-sensitive stroke treatment, particularly in hospitals where specialist expertise would otherwise not be immediately available. However, the observed difference in treatment rates may reflect several mechanisms, including improved access to vascular neurology expertise, more efficient patient selection, strengthened acute stroke protocols, and increased confidence among local clinicians.

Implementation studies provide further evidence that treatment delivery changes after the introduction of telemedicine-supported stroke services. Nguyen-Huynh et al. reported that implementation of a telestroke program across 21 hospitals within an integrated healthcare system was associated with improved delivery of thrombolytic therapy (11). More recent observational work has similarly shown improvement in acute stroke protocol performance following the introduction of neurological assessment by telemedicine (10). At the health-system level, Janssen et al. found that support from telestroke networks was associated not only with increased use of intravenous thrombolysis but also with reduced interhospital transfer, suggesting that remote specialist support may enable more patients to receive treatment at the presenting hospital (9).

Meta-analytic evidence also supports an overall association between telemedicine-supported stroke care and increased reperfusion treatment. Recent syntheses of telestroke and telemedicine-administered thrombolysis have generally reported higher treatment utilization or improved access to thrombolytic therapy in hospitals supported by remote stroke expertise (6, 22). Nevertheless, substantial heterogeneity exists across telestroke networks with respect to referral pathways, staffing models, imaging availability, transfer practices, treatment eligibility, and the denominator used to calculate thrombolysis rates. Direct comparison of absolute treatment percentages across studies should therefore be interpreted cautiously.

Taken together, the available evidence supports a relatively robust conclusion: telestroke is associated with increased access to and utilization of reperfusion therapy. What remains uncertain is the mechanism underlying this association. Existing studies were generally designed to assess treatment delivery, workflow, or clinical outcomes rather than clinicians’ diagnostic confidence or treatment thresholds. Accordingly, the higher thrombolysis rates observed in telestroke systems cannot determine whether more patients are treated because previously undertreated eligible patients gain access to specialist care, because stroke pathways become more efficient, because selection practices differ, or because clinicians become more willing to treat diagnostically uncertain cases. This distinction is central to the threshold-shift hypothesis examined later in this review.

The principal clinical evidence demonstrating the association between telestroke implementation and greater thrombolysis utilization is summarized in Figure 2.

**Figure 2:**
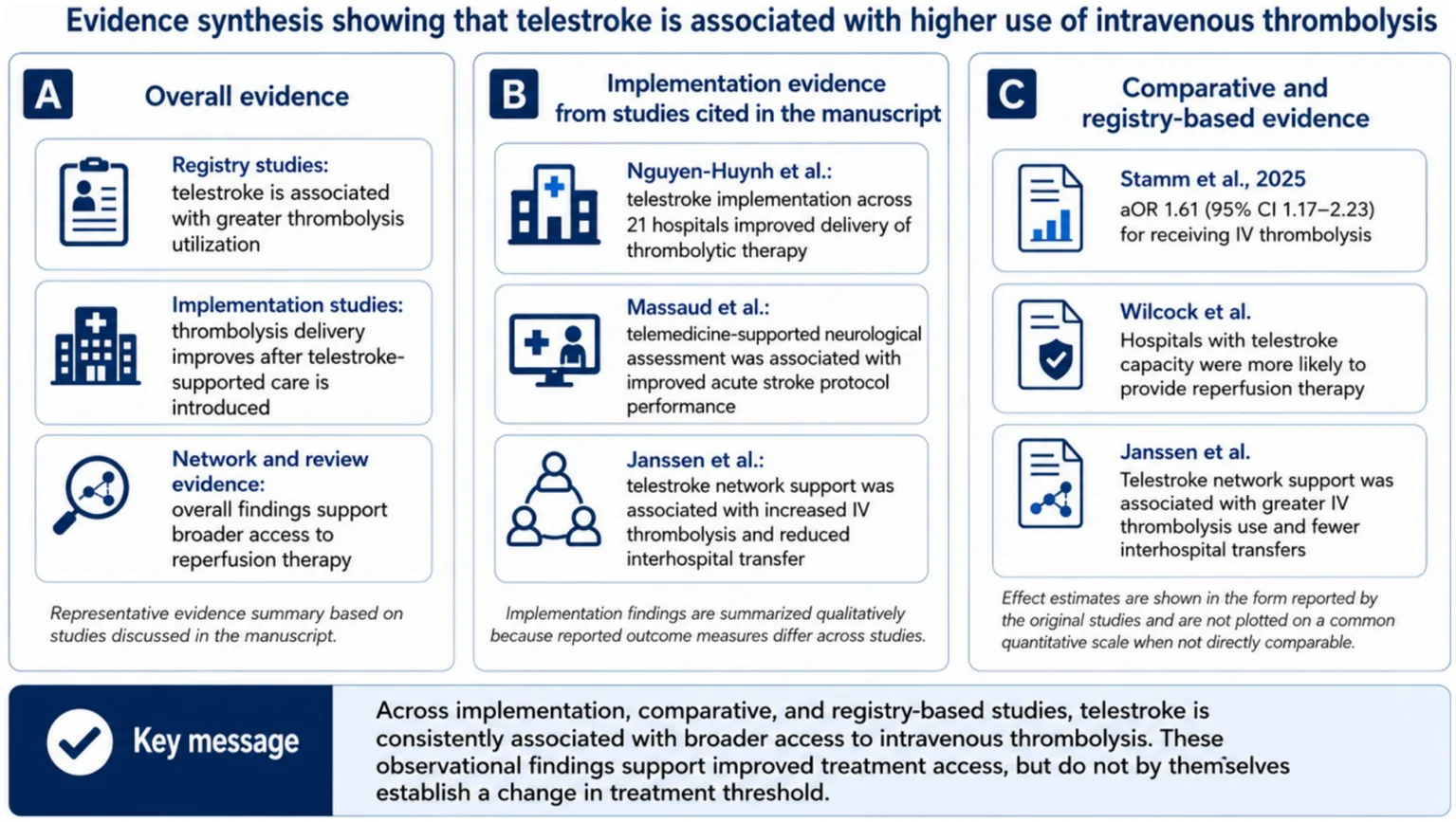
Evidence synthesis of thrombolysis utilization in telestroke-supported care. **(A)** Overall evidence from registry, implementation, and review studies indicates that telestroke is associated with greater use of intravenous thrombolysis and broader access to reperfusion therapy. **(B)** Representative implementation studies cited in the manuscript illustrate improvements in thrombolytic treatment delivery and acute stroke protocol performance, together with increased IV thrombolysis and reduced interhospital transfer following the introduction or expansion of telestroke-supported care. Findings in panels A and B are summarized qualitatively because study populations, denominators, and reported outcome measures differ across studies. **(C)** Comparative and registry-based evidence further supports an association between telestroke availability and greater reperfusion treatment utilization. The adjusted odds ratio reported by Stamm et al. is shown in the metric reported by the original study, whereas findings from other studies are presented qualitatively rather than on a common quantitative scale because their effect measures are not directly comparable. Collectively, these findings support improved treatment access associated with telestroke but do not, by themselves, establish a change in the clinical threshold for thrombolysis. IVT, intravenous thrombolysis; aOR, adjusted odds ratio; CI, confidence interval.

### 3.2 Stroke mimics in telestroke practice

Stroke mimics are non-vascular conditions that produce acute neurological symptoms resembling ischemic stroke. Common etiologies include epileptic seizures, migraine, functional neurological disorders, toxic or metabolic disturbances, and peripheral vestibular disorders (12). Distinguishing these conditions from true cerebral ischemia can be difficult during hyperacute assessment, particularly before diagnostic investigations and clinical evolution clarify the underlying cause. In a comprehensive review including more than 60 studies and over 62,000 patients evaluated for suspected ischemic stroke, Pohl et al. estimated that 24.8% were ultimately diagnosed with stroke mimics. The most frequently reported etiologies included peripheral vestibular disorders, toxic or metabolic conditions, seizures, functional neurological disorders, and migraine (13).

The frequency of stroke mimics reported in telestroke studies varies substantially, in part because different studies evaluate different patient populations. Some cohorts include all patients referred for remote stroke assessment, whereas others include only patients selected for intravenous thrombolysis. This distinction is important when interpreting apparently different mimic rates. In a large telestroke consultation cohort, Chhabra et al. evaluated 1,043 patients and reported that 36.5% were ultimately classified as stroke mimics (23). Such all-comer consultation cohorts characterize the degree of diagnostic uncertainty encountered before treatment selection and should not be directly compared with studies limited to patients who actually received thrombolysis.

Among thrombolysis-treated patients, Lee et al. examined 270 patients who received intravenous thrombolysis following telestroke evaluation and found that 64 patients (23.7%) were subsequently classified as stroke mimics (14). Importantly, no symptomatic intracranial hemorrhage was reported among the mimic patients in that cohort. The study therefore illustrates that thrombolysis may be administered while diagnostic uncertainty remains.

Comparative studies provide additional context. Sinn et al. reported a stroke mimic proportion of 27.2% among thrombolysed patients managed through a telestroke pathway, compared with 14.8% among patients treated in-house (15). This finding is compatible with greater treatment of diagnostically uncertain presentations in the telestroke pathway, but several alternative explanations remain possible, including differences in referral patterns, patient selection, imaging availability, local expertise, and diagnostic adjudication. An earlier comparison by Yaghi et al. likewise reported a higher proportion of thrombolysed stroke mimics in a telestroke setting than in conventional primary stroke center care (24).

Not all studies, however, have reported similarly high rates. Nair et al. examined thrombolysis across comprehensive stroke centers and telestroke-dependent primary stroke centers and reported a mimic thrombolysis rate of approximately 5.36% at telestroke sites, compared with approximately 4.3% in the comparator setting (25). Their analysis used more stringent chart- and imaging-supported classification to identify true stroke mimics. The marked difference between these estimates and those reported in single-center telestroke series illustrates an important methodological issue: the apparent frequency of stroke mimics depends strongly on how the study population is defined and how the final diagnosis is adjudicated. For this reason, mimic percentages across telestroke studies should not be interpreted as directly interchangeable measures of diagnostic error.

The clinical relevance of stroke mimics also depends on the consequences of thrombolysis in patients ultimately diagnosed with stroke mimics. Available evidence has generally shown that symptomatic intracranial hemorrhage is uncommon among patients ultimately diagnosed with stroke mimics. In a prospective study and meta-analysis, Tsivgoulis et al. found a substantially lower hemorrhagic risk among thrombolysed stroke mimics than among patients with confirmed ischemic stroke (17) Similarly, analyses from the NOR-TEST population reported no symptomatic intracranial hemorrhage among stroke mimics receiving thrombolytic treatment (16). Large registry evidence has also demonstrated that serious hemorrhagic complications occur infrequently in thrombolysed mimics (18). These observations support the relatively favorable hemorrhagic safety profile of thrombolysis in appropriately screened patients ultimately diagnosed with stroke mimics, while recognizing that such treatment may still carry clinical, economic, or organizational consequences.

Taken together, the literature supports three conclusions. First, stroke mimics constitute a substantial component of acute stroke evaluation, including within telestroke systems. Second, the reported frequency varies considerably according to whether the denominator consists of all suspected stroke consultations or only thrombolysis-treated patients and according to how the final diagnosis is established. Third, severe hemorrhagic complications following thrombolysis of stroke mimics appear to be uncommon. These findings define an important context for telestroke decision-making under uncertainty.

The epidemiology of stroke mimics and representative telestroke cohorts are summarized in Figure 3 and Table 1.

**Figure 3:**
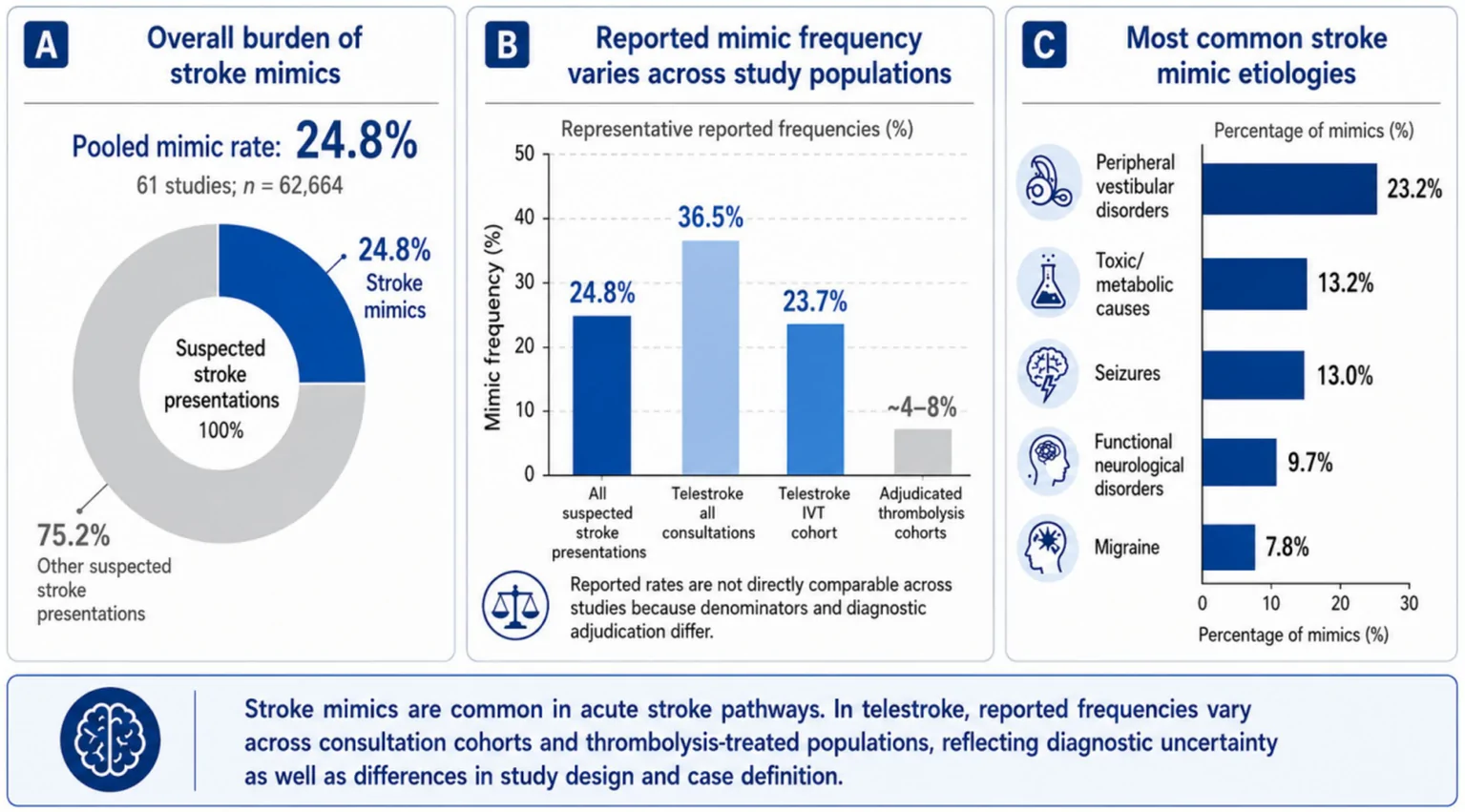
Stroke mimic burden and heterogeneity across acute stroke evaluation settings. **(A)** Pooled analyses indicate that stroke mimics constitute a substantial proportion of patients evaluated for suspected acute stroke. **(B)** Representative mimic frequencies vary across all-comer stroke populations, telestroke consultation cohorts, thrombolysis-treated telestroke cohorts, and more stringently adjudicated thrombolysis cohorts. These estimates are not directly comparable because denominators, patient selection, and methods of final diagnostic adjudication differ. **(C)** Common stroke mimic etiologies include peripheral vestibular disorders, toxic or metabolic conditions, seizures, functional neurological disorders, and migraine. The figure illustrates both the clinical importance of stroke mimics and the methodological heterogeneity that limits simple comparisons between telestroke and conventional care.

**Table 1:** Representative studies of stroke mimics relevant to telestroke thrombolysis and safety.

Study	Population and setting	*N*	Stroke mimic ascertainment	Mimic rate	Hemorrhagic outcome in mimics	Main finding
(14)	IV thrombolysis after telestroke evaluation; US academic hub-and-spoke network	270	Final diagnosis after clinical work-up; imaging and alternative diagnosis considered	23.7% (64/270)	sICH 0%	Nearly one quarter of thrombolysed telestroke patients were ultimately classified as stroke mimics.
(15)	IVT-treated patients; statewide telestroke pathway versus in-house treatment	195 telestroke; 88 in-house	24-h imaging, discharge diagnosis, and neurologist panel adjudication	27.2% vs 14.8%	Any ICH among mimics: 3.0% overall; sICH not separately reported	Mimic frequency was higher in the telestroke group than in the in-house group (*p* = 0.033), although methodology and case selection may contribute.
(26)	Thrombolytic-treated patients evaluated by telemedicine in a rural US stroke network	171	Final diagnosis categorized as cerebrovascular disease (AIS/TIA) or stroke mimic after chart review	25.0% (43/171)	NR separately for sICH	Stroke mimics accounted for one quarter of treated patients; the TeleStroke Mimic Score showed limited discrimination.
(25)	Thrombolysis at telestroke-dependent primary stroke centers versus comprehensive stroke centers; Alberta, Canada	2,471 total; 503 treated at telestroke PSCs	Registry linkage followed by detailed clinical, imaging, electronic-record, and PACS review	5.36% telestroke PSC vs 4.3% CSC	1/112 true mimics (0.89%) had a small parenchymal hemorrhage; sICH not separately reported	No significant difference in mimic thrombolysis between telestroke PSCs and CSCs; rigorous adjudication substantially reduced the apparent mimic rate.
(24)	IV tPA through a statewide telestroke network versus conventional primary stroke center	252 treated overall	Final non-stroke diagnosis after clinical evaluation; telestroke patients remaining at peripheral hospitals were excluded from diagnostic comparison	7.8% telestroke vs 4.3% conventional	NR	Mimic proportions were not significantly different between telestroke and conventional care (*p* = 0.53).
(23)	All-comer suspected acute stroke patients undergoing video evaluation in a large academic telestroke network	1,043	Final imaging and discharge diagnosis used to classify cerebral ischemia versus mimic	36.5% (381/1,043)	NR	Stroke mimics represented more than one third of all telestroke evaluations; existing mimic scores had insufficient performance for standalone clinical use.
(17)	Prospective IVT-treated cohort plus meta-analysis; non-telestroke safety context	516 prospective patients; 75 mimics	Final diagnosis after diagnostic work-up	14.5% (75/516)	Prospective cohort: 1.3% (1/75) sICH; pooled meta-analysis: 0.5% sICH	Provides prospective and pooled evidence that serious hemorrhage after IVT in stroke mimics is uncommon.
(16)	NOR-TEST randomized thrombolysis population; tenecteplase or alteplase	1,091	Discharge diagnosis other than ischemic stroke or TIA	16.6% (181/1,091)	sICH 0%	Trial-based evidence supports a favorable hemorrhagic safety profile of thrombolysis in stroke mimics.
(18)	Get With The Guidelines–Stroke registry; US tPA-treated patients	72,582; 2,517 mimics	Stroke-like presentation followed by work-up showing neither stroke nor TIA	3.5%	sICH 0.4% vs 3.5% in ischemic stroke	Large registry evidence confirms that serious hemorrhagic complications are substantially less frequent in mimics than in true ischemic stroke.

Studies are grouped according to their relevance to telestroke-specific practice and the broader safety of thrombolysis in stroke mimics. Reported mimic rates are not directly comparable across studies because denominators, clinical settings, and methods of final diagnostic adjudication differ. Hemorrhagic outcomes are presented as reported in the original studies; not all studies used the same definition of symptomatic intracranial hemorrhage. AIS, acute ischemic stroke; CSC, comprehensive stroke center; ICH, intracranial hemorrhage; IVT, intravenous thrombolysis; NR, not reported; PACS, picture archiving and communication system; PSC, primary stroke center; sICH, symptomatic intracranial hemorrhage; TIA, transient ischemic attack.

### 3.3 Evidence relevant to treatment under diagnostic uncertainty

Several findings from the available literature are relevant to understanding how thrombolysis decisions are made when the diagnosis remains uncertain. First, telestroke-supported care is consistently associated with greater use of intravenous thrombolysis across registry, implementation, and comparative studies (7–11). Second, stroke mimics are encountered among both patients referred for telestroke evaluation and those who ultimately receive thrombolysis (14, 15, 23–25). Third, serious hemorrhagic complications among thrombolysed stroke mimics appear to be uncommon in published cohorts (16–18).

These observations demonstrate that treatment decisions in acute stroke care may occur before diagnostic certainty is complete. They also show that the consequences of diagnostic uncertainty are not necessarily symmetrical: failure to treat a true ischemic stroke may result in loss of a time-sensitive therapeutic opportunity, whereas thrombolysis in a patient ultimately diagnosed with a mimic is associated with a comparatively low rate of symptomatic intracranial hemorrhage (17, 18, 27).

However, none of these findings directly measures clinicians’ treatment thresholds. Increased thrombolysis utilization may result from improved access to specialists, more efficient workflows, greater adherence to treatment protocols, or changes in patient selection. Similarly, a higher proportion of treated stroke mimics may reflect differences in referral patterns, case mix, or diagnostic adjudication rather than a change in clinicians’ willingness to treat uncertain cases.

Therefore, the current evidence supports the presence of treatment under diagnostic uncertainty, but does not establish that telestroke lowers the probability of ischemic stroke required for thrombolysis. Whether telestroke modifies this practical treatment threshold is addressed as a hypothesis in the Discussion.

## 4 Discussion

### 4.1 Interpreting the threshold-shift hypothesis

The evidence summarized in this review supports a clear conclusion that telestroke expands access to reperfusion therapy and is associated with greater use of intravenous thrombolysis (7–9). Stroke mimics are also encountered with meaningful frequency in telestroke pathways, including among patients who receive thrombolysis (14, 15, 24, 25). These observations raise an important question: does telestroke simply improve access to treatment, or might it also influence the level of diagnostic certainty at which treatment is considered acceptable?

The distinction is important because a treatment threshold is not equivalent to a treatment rate. In this review, the practical thrombolysis threshold refers to the level of diagnostic confidence at which the expected benefit of immediate treatment is judged to outweigh the potential harm of thrombolysis and the cost of further diagnostic delay. A higher thrombolysis rate does not necessarily indicate a lower threshold. It may instead reflect improved access to specialists, faster workflows, better recognition of eligible patients, or stronger adherence to established treatment protocols.

For the same reason, the treatment of stroke mimics should not be regarded as direct evidence of threshold change. A patient may ultimately receive a non-stroke diagnosis despite having had a clinically reasonable probability of ischemic stroke at the time thrombolysis was administered. Final diagnosis is determined retrospectively, whereas treatment decisions are made prospectively with incomplete information. Mimic treatment therefore demonstrates that decisions sometimes occur under uncertainty, but it does not reveal how much uncertainty clinicians were willing to accept.

The threshold-shift hypothesis is instead based on the possibility that telestroke creates a somewhat different decision environment. Remote assessment may involve time pressure, incomplete or mediated clinical information, and shared decision-making between hub and spoke teams (4, 20, 28, 29). At the same time, the consequences of treatment delay and the relatively low rate of serious hemorrhagic complications reported in thrombolysed stroke mimics create an asymmetric clinical risk structure (16–18, 27). These features provide plausible mechanisms through which treatment behavior might differ, but they do not establish that such a difference actually occurs.

Alternative explanations remain equally important. Telestroke may primarily correct previous undertreatment at hospitals without immediate neurological expertise, improve protocol adherence, reduce delays, and allow treatment to occur locally rather than after transfer (8–11). Differences in case mix, hospital resources, network maturity, and diagnostic adjudication can also influence both thrombolysis rates and observed mimic frequency. Current observational studies cannot adequately separate these effects from a true change in treatment threshold.

Accordingly, we use threshold shift as a hypothesis-generating framework rather than a causal conclusion. Demonstrating a threshold effect would require direct measurement of clinicians’ diagnostic confidence or estimated probability of ischemic stroke at the time treatment is recommended. Until such evidence is available, increased thrombolysis utilization and mimic treatment should be interpreted as indirect observations that motivate investigation of decision thresholds rather than as proof that those thresholds have changed.

The conceptual relationship between the telestroke decision environment and the proposed threshold-shift hypothesis is illustrated in Figure 4.

**Figure 4:**
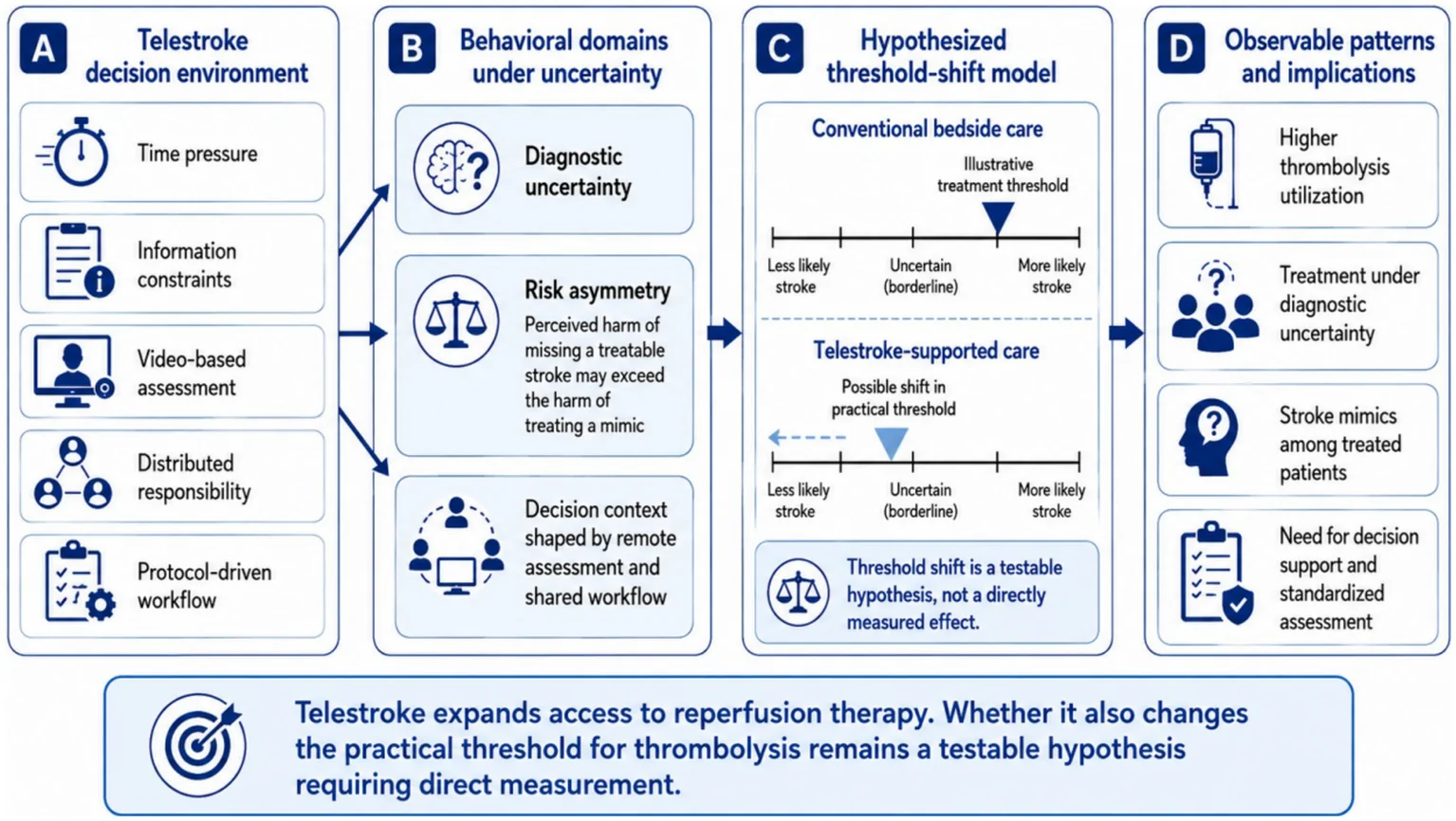
Conceptual model of the hypothesized treatment-threshold shift in telestroke. **(A)** The telestroke decision environment is shaped by time pressure, information constraints, video-based assessment, distributed responsibility, and protocol-driven workflow. **(B)** These features may influence decision-making through diagnostic uncertainty, risk asymmetry, and the organization of remote team-based care. **(C)** The model illustrates a possible change in the practical probability or level of diagnostic confidence at which thrombolysis is considered appropriate in telestroke-supported care compared with conventional bedside care. The illustrated threshold positions are conceptual and do not represent empirically measured values. **(D)** Observed patterns relevant to this hypothesis include greater thrombolysis utilization, treatment under diagnostic uncertainty, and the occurrence of stroke mimics among treated patients, together with a need for standardized assessment and decision support. The threshold-shift model is hypothesis-generating and requires direct prospective measurement of diagnostic confidence and treatment decisions.

### 4.2 Risk asymmetry and treatment under uncertainty

A central feature of thrombolysis decision-making is the unequal consequence of competing errors. In this review, risk asymmetry refers to the difference between the potential harm of withholding or delaying thrombolysis in a patient with true acute ischemic stroke and that of treating a patient who is subsequently diagnosed with a stroke mimic. Because the benefit of reperfusion is time dependent, delayed treatment may result in irreversible neurological injury, whereas serious hemorrhagic complications among appropriately screened stroke mimics are comparatively uncommon (16–18, 27).

This imbalance creates a setting in which some degree of diagnostic uncertainty may be accepted before treatment. The decision is not simply whether the diagnosis is completely certain, but whether the expected benefit of immediate reperfusion outweighs the combined risks of treatment and further delay. When the potential consequence of missing a treatable ischemic stroke is perceived to be greater than the consequence of treating a patient ultimately diagnosed with a mimic, clinicians may reasonably favor action despite residual uncertainty.

Risk asymmetry, however, is not specific to telestroke. The same trade-off exists in conventional bedside care. Its relevance to telestroke lies in the possibility that remote assessment, time pressure, and reliance on information transmitted through the spoke team may alter how these competing risks are weighed (4, 20, 29). Current studies do not directly compare the perceived consequences of withholding treatment and treating patients later diagnosed with stroke mimics between telestroke and bedside clinicians, and therefore cannot establish that telestroke increases risk tolerance.

This distinction is also important when interpreting stroke mimics. A final mimic diagnosis does not necessarily indicate that the original treatment decision was inappropriate. Thrombolysis is given prospectively, whereas the final diagnosis is often established only after further imaging, observation, or clinical evolution. Decision quality should therefore be judged according to the information and estimated probability of stroke available at the time of treatment rather than by final diagnosis alone.

Whether risk asymmetry contributes differently to thrombolysis decisions in telestroke and conventional care remains uncertain and requires prospective evaluation.

### 4.3 Information constraints and diagnostic friction in remote assessment

A second behavioral domain concerns the information available at the time of treatment. We use information constraints to describe limitations in the completeness or reliability of clinical information during remote evaluation, while diagnostic friction refers to the difficulty of resolving these uncertainties within a time-sensitive treatment pathway.

Telestroke examination is generally feasible and clinically reliable. Remote NIHSS assessment has shown good overall agreement with bedside evaluation, and subsequent studies have supported the diagnostic utility of teleneurological consultation (20, 28, 30, 31). Nevertheless, some elements of the examination and clinical context may be more difficult to assess remotely. Subtle coordination abnormalities, inconsistent symptoms, patient cooperation, collateral history, baseline neurological status, and the temporal evolution of symptoms may all influence the differential diagnosis but may not be equally accessible during a video consultation. Telestroke guidelines therefore emphasize trained bedside personnel, structured communication, and standardized examination procedures (4, 29, 32).

These limitations are particularly relevant when evaluating stroke mimics. Migraine, seizures, functional neurological disorders, metabolic disturbances, and other mimic conditions may overlap substantially with ischemic stroke at presentation (12, 13). In many cases, diagnostic certainty improves only after additional history, repeat examination, imaging, or observation. In hyperacute stroke care, however, further diagnostic clarification competes directly with the need to avoid treatment delay (27, 33).

This interaction between incomplete information and limited time constitutes diagnostic friction. Importantly, its behavioral effect is not necessarily unidirectional. Residual uncertainty could favor treatment when clinicians prioritize avoiding a missed stroke, but it could also promote caution when the available information is considered insufficient. Existing studies do not establish which response predominates.

Information constraints should therefore be viewed as a potential modifier of decision-making rather than evidence of a lower treatment threshold. Their relevance to the present hypothesis is that telestroke may alter the relationship between diagnostic confidence, available information, and treatment timing; whether this changes treatment behavior remains uncertain.

### 4.4 Distributed responsibility and workflow pressure

Telestroke decisions are inherently collaborative. In this review, distributed responsibility refers to the sharing of diagnostic assessment and treatment decisions between the remote stroke specialist and the local bedside team, whereas workflow pressure refers to the organizational emphasis on rapid evaluation and timely reperfusion. Telestroke guidelines consequently emphasize clear role allocation, standardized communication, trained spoke personnel, and predefined treatment pathways (4, 29, 32).

This structure may influence how uncertainty is managed. The remote consultant relies on the spoke team for examination findings, history, vital signs, and treatment execution, while local clinicians depend on specialist interpretation and recommendations. Ethical and organizational literature on teleneurology has therefore highlighted accountability, documentation, communication, and role clarity as important components of remote care (34). At the same time, telestroke systems are specifically designed to reduce treatment delays, and their effectiveness depends heavily on workflow standardization and coordination across participating hospitals (33, 35, 36).

The behavioral consequences of these features remain uncertain. Shared responsibility could theoretically favor treatment when clinicians are particularly concerned about missing an eligible ischemic stroke, but it could also increase caution when either the hub or spoke clinician remains uncomfortable with the diagnosis. Likewise, protocol-driven workflows may accelerate treatment, yet standardization may also improve diagnostic consistency and treatment calibration. Current evidence therefore does not support the assumption that distributed responsibility or workflow pressure necessarily lowers the thrombolysis threshold.

Network maturity may further modify these effects. As teams gain experience, communication and examination procedures may become more consistent, allowing uncertainty to be managed more efficiently (32, 36).

Overall, distributed responsibility and workflow pressure should be considered features of the telestroke decision environment rather than established causes of greater thrombolysis utilization. Their potential contribution to a threshold shift remains a testable hypothesis. The three behavioral domains proposed in this review, together with their operational definitions, telestroke-specific contexts, potential influences on decision-making, and current evidence status, are summarized in Table 2.

**Table 2:** Proposed behavioral framework for thrombolysis decision-making under uncertainty in telestroke.

Behavioral domain	Operational definition	Telestroke-specific context	Potential influence on decision-making	Evidence status
Risk asymmetry	Unequal perceived consequences of withholding or delaying IVT in true ischemic stroke versus administering IVT to a patient later diagnosed with a stroke mimic.	Reperfusion benefit is time dependent, whereas serious hemorrhagic complications after thrombolysis of stroke mimics are uncommon (16–18, 27).	May increase willingness to treat despite residual diagnostic uncertainty when the perceived consequence of missing a treatable stroke exceeds that of treating a mimic.	The clinical asymmetry is supported by thrombolysis and mimic-safety data, but its effect on clinicians’ treatment thresholds has not been directly measured.
Information constraints and diagnostic friction	Limitations in the completeness, reliability, or immediacy of information available during remote assessment, together with the difficulty of resolving uncertainty within a narrow treatment window.	Telestroke relies on video examination, locally obtained history and examination findings, imaging, and communication between the spoke team and remote consultant (4, 20, 28, 29).	Residual uncertainty may favor rapid treatment in some cases, but may increase caution in others; the direction of effect is likely context dependent.	Remote neurological assessment is generally feasible and reliable, but there is no direct evidence that information constraints systematically lower the treatment threshold (20, 28, 30, 31).
Distributed responsibility and workflow pressure	Sharing of diagnostic assessment and treatment responsibility between hub and spoke clinicians within a workflow designed to minimize treatment delay.	Treatment decisions depend on communication, role allocation, protocolized assessment, and coordination across participating hospitals (4, 29, 32, 33, 35, 36).	Shared responsibility and time-oriented workflows may alter how uncertainty is negotiated, although standardization may also improve consistency and improve treatment calibration.	Organizational and implementation evidence supports the relevance of these factors, but their independent effect on thrombolysis decisions remains hypothesized rather than established.

The three domains were derived through narrative synthesis of recurrent decision-related themes in the telestroke and acute stroke literature and are proposed as a conceptual framework rather than a validated behavioral taxonomy. The potential influences listed are hypothesis-generating and should not be interpreted as evidence that telestroke lowers the thrombolysis treatment threshold. IVT, intravenous thrombolysis; NIHSS, National Institutes of Health Stroke Scale.

### 4.5 Alternative explanations and limitations of the threshold-shift hypothesis

Several alternative explanations must be considered before attributing higher thrombolysis utilization in telestroke systems to a change in treatment threshold. Most importantly, telestroke was designed to correct limitations in access to specialist stroke care. Hospitals without on-site neurological expertise may have historically treated fewer eligible patients because of diagnostic uncertainty, delays in specialist consultation, or the need for transfer. By providing immediate access to vascular neurology expertise, telestroke may therefore increase thrombolysis simply by reducing previous undertreatment rather than by changing the level of diagnostic certainty considered sufficient for treatment (8–11).

Differences in case mix and service organization provide additional sources of confounding. Telestroke networks vary in referral criteria, hospital resources, imaging availability, clinician experience, transfer practices, and the populations served. Some networks predominantly support small rural hospitals, whereas others operate within integrated stroke systems with established local protocols. These differences may affect both treatment rates and the types of patients referred for remote consultation (4, 29, 32, 36). Temporal improvements in stroke pathways, broader implementation of reperfusion protocols, and increasing network maturity may further increase treatment utilization independently of any behavioral change.

The interpretation of stroke mimic data is subject to similar limitations. Reported mimic frequencies differ substantially according to whether studies include all telestroke consultations or only thrombolysis-treated patients, and according to how the final diagnosis is established. For example, comparatively high mimic proportions have been reported in some telestroke thrombolysis cohorts, whereas studies using more restrictive chart- and imaging-based adjudication have produced substantially lower estimates (14, 15, 23–25). Mimic frequency therefore cannot be used as a standardized surrogate for treatment threshold across studies.

A more fundamental limitation is that existing evidence rarely measures the treatment threshold itself. Most available studies are observational and report thrombolysis utilization, workflow performance, final diagnoses, or clinical outcomes (7–9, 22). They generally do not record clinicians’ estimated probability of ischemic stroke, degree of diagnostic confidence, competing diagnoses, or the level of uncertainty considered acceptable immediately before treatment. Consequently, the central behavioral mechanism proposed in this review cannot currently be distinguished from improved access, more efficient care, or differences in patient selection.

The behavioral framework developed here also has limitations. Risk asymmetry, information constraints, and distributed responsibility were selected as recurrent and clinically relevant themes rather than as components of a previously validated taxonomy. Their proposed effects may overlap and may differ across clinicians, hospitals, and network configurations. They should therefore be regarded as hypothesis-generating constructs, not established determinants of telestroke treatment behavior.

Finally, the present review is narrative rather than systematic. Although priority was given to telestroke-specific cohorts, large registries, meta-analyses, and studies with clearly described diagnostic methods, heterogeneity in study design prevents quantitative estimation of a threshold effect. The available literature supports the possibility that telestroke changes how uncertainty is managed, but it does not establish that such a change occurs or determine its direction or magnitude. This uncertainty should be viewed as a key evidence gap rather than as a weakness of the conceptual question itself.

## 5 Clinical implications and future research

The practical implication of the threshold-shift hypothesis is not that telestroke should become more restrictive in the use of thrombolysis. The principal benefit of telestroke remains its ability to extend specialist stroke care to hospitals where such expertise would otherwise be unavailable. The more relevant objective is to improve how diagnostic uncertainty is managed without sacrificing the speed that makes reperfusion effective. Future strategies should therefore seek to increase diagnostic confidence, support rather than replace clinical judgment, and measure decision behavior more directly.

### 5.1 Improving diagnostic certainty without delaying treatment

Imaging and structured remote assessment offer complementary opportunities to reduce uncertainty during telestroke consultation. Automated imaging tools can assist with the detection of large-vessel occlusion, early ischemic change, and perfusion abnormalities, and several systems have demonstrated promising diagnostic performance in acute stroke workflows (37–41). More importantly for telestroke, prospective evaluation within a large telestroke network has shown the feasibility of integrating artificial intelligence-based imaging support into remote acute stroke assessment (42).

The value of such technologies should not be judged solely by diagnostic accuracy. A tool that improves image interpretation but delays treatment may offer little practical advantage in hyperacute stroke care. Conversely, rapid decision support may be useful if it reduces uncertainty in borderline cases without creating additional workflow complexity. Future studies should therefore evaluate effects on treatment time, diagnostic agreement, treatment selection, and clinical outcomes rather than technical performance alone.

Standardization of the remote examination is equally important. Telestroke guidelines and contemporary organizational recommendations emphasize structured neurological assessment, trained bedside personnel, reliable audiovisual communication, and clearly defined hub–spoke workflows (4, 29, 32). These measures may reduce avoidable variation in the information available to the consultant. They are particularly relevant in cases in which subtle examination findings or incomplete history contribute to uncertainty.

The aim should therefore be to improve diagnostic certainty per unit of time, rather than to pursue diagnostic certainty at the expense of timely reperfusion. This distinction is central to any strategy intended to improve decision quality in telestroke.

### 5.2 Stroke mimic prediction and decision support

Clinical prediction tools represent another potential approach to managing uncertainty. The TeleStroke Mimic Score and subsequent validation studies were developed specifically to estimate the probability that a patient evaluated through telestroke has a stroke mimic (43, 44). More recent work, however, suggests that currently available mimic scores may have limited discriminatory performance when applied across large and heterogeneous telestroke populations (23).

These findings argue against using a mimic score as an isolated rule for withholding thrombolysis. A patient classified as having a relatively high probability of a mimic may still have a clinically important ischemic stroke, and rigid application of a prediction model could introduce harmful treatment delay or false-negative decisions. Prediction tools may therefore be more useful as decision-support instruments that prompt targeted reconsideration of alternative diagnoses rather than as treatment exclusion criteria.

Future models could integrate clinical variables with imaging findings, symptom evolution, previous medical history, and features of the remote examination. Their evaluation should extend beyond conventional discrimination measures. Clinically relevant outcomes include the frequency of thrombolysis among patients ultimately diagnosed with stroke mimics, missed or delayed treatment of true ischemic stroke, and how clinicians use the information under time pressure.

Decision support should therefore aim for better calibrated treatment selection rather than simply minimizing the number of treated stroke mimics.

### 5.3 Direct measurement of treatment thresholds

The most important research gap identified by this review is the absence of direct measurement of the thrombolysis treatment threshold. Current studies primarily report whether treatment was given, how rapidly it was administered, and what diagnosis was eventually established. These outcomes cannot determine how much diagnostic certainty clinicians required when the decision was actually made.

Future studies should therefore capture decision variables prospectively. Before the final diagnosis is known, clinicians could record their estimated probability of ischemic stroke, degree of diagnostic confidence, leading alternative diagnoses, perceived risk of withholding treatment, and reasons for recommending or declining thrombolysis. These measurements could then be related to treatment decisions and final clinical outcomes.

Controlled approaches may provide even stronger evidence. Standardized clinical vignettes or simulated stroke encounters could present the same clinical information to telestroke and bedside clinicians and compare the probability of stroke at which thrombolysis is recommended. Alternatively, matched observational studies could compare patients with similar neurological deficits, imaging findings, treatment windows, and comorbidities across remote and in-person settings. Such designs would help distinguish a true decision-threshold effect from differences in access, case mix, or workflow.

Threshold behavior should also be examined over time. Mature telestroke networks may differ from newly established programs because clinicians and spoke teams develop more efficient communication, greater familiarity with local resources, and better calibration of diagnostic uncertainty. Longitudinal evaluation could therefore determine whether treatment decisions become more consistent as network experience increases.

Importantly, any future measure of treatment threshold should be interpreted alongside patient-centered outcomes. A lower threshold would not necessarily be harmful if it increased treatment of true ischemic stroke without a meaningful increase in serious adverse events. Conversely, a higher threshold would not necessarily indicate better diagnostic performance if it resulted in missed reperfusion opportunities. Future research should therefore assess diagnostic confidence, treatment probability, door-to-needle time, mimic treatment, missed ischemic stroke, hemorrhagic complications, and functional outcome together, rather than treating any single metric as evidence of decision quality.

The key objective is not to eliminate uncertainty from telestroke—an unrealistic goal in hyperacute stroke care—but to make that uncertainty measurable, transparent, and better calibrated to treatment decisions. The principal research and implementation priorities for improving thrombolysis decision-making under uncertainty in telestroke are summarized in Table 3.

**Table 3:** Research and implementation priorities for thrombolysis decision-making in telestroke.

Priority	Proposed approach	Rationale	Current evidence	Key outcomes for future evaluation
Improve diagnostic certainty without delaying treatment	Integrate rapid imaging support, including automated assessment of early ischemic change, large-vessel occlusion, and selected perfusion abnormalities, into existing telestroke workflows.	Additional diagnostic information may help resolve uncertainty in borderline presentations, but any benefit must be balanced against treatment delay.	AI-assisted and advanced imaging approaches have shown feasibility and potential utility in acute stroke and telestroke workflows (19, 37–42, 45). Evidence that these tools improve thrombolysis selection remains limited.	Diagnostic agreement; door-to-needle time; treatment modification; missed ischemic stroke; thrombolysis among patients ultimately diagnosed with stroke mimics; functional outcome.
Improve recognition of stroke mimics	Use validated clinical prediction tools as adjuncts to, rather than substitutes for, clinical assessment; develop models integrating clinical, imaging, and temporal features.	Mimic prediction may help identify cases requiring closer diagnostic review without using a score as an automatic reason to withhold IVT.	The TeleStroke Mimic Score has been developed and externally evaluated (43, 44, 46). Recent telestroke data show limited discrimination of existing scores (23).	Discrimination and calibration; false-negative ischemic strokes; mimic thrombolysis; treatment delay; clinician uptake.
Standardize remote assessment and hub–spoke workflow	Use structured neurological examination, standardized information transfer, trained spoke personnel, clear role allocation, and predefined escalation pathways.	Reducing avoidable variation in information quality and communication may improve consistency while preserving rapid treatment.	Supported by telestroke guidelines, remote examination studies, and implementation research (4, 28, 29, 32, 33, 36).	Examination completeness; hub–spoke agreement; consultation time; door-to-needle time; protocol adherence; diagnostic concordance.
Measure treatment thresholds directly	Prospectively record pre-treatment probability of ischemic stroke, clinician confidence, competing diagnoses, and reasons for treatment or non-treatment; complement observational studies with standardized vignettes or matched remote-versus-bedside comparisons.	Treatment rate and final diagnosis cannot determine the level of diagnostic certainty required for treatment. Direct measurement is necessary to distinguish an access effect from a true decision-threshold effect.	Major evidence gap. Existing telestroke studies largely measure treatment utilization, workflow, final diagnosis, and outcomes rather than the decision threshold itself (7–9, 14).	Estimated stroke probability at treatment; diagnostic confidence; treatment recommendation; threshold variation across settings and clinicians; clinical outcomes.

Proposed strategies are intended to improve the calibration of treatment decisions while preserving timely reperfusion. Reduction in stroke mimic thrombolysis should not be considered beneficial if it increases delayed or missed treatment of true ischemic stroke. Direct measurement of diagnostic confidence and estimated stroke probability is required to distinguish improved treatment access from a true decision-threshold effect. AI, artificial intelligence; IVT, intravenous thrombolysis.

## 6 Conclusion

Telestroke has become an important component of contemporary stroke systems by extending specialist expertise to hospitals where immediate neurological assessment may otherwise be unavailable. The available literature consistently associates telestroke with broader access to reperfusion therapy and greater use of intravenous thrombolysis. Stroke mimics are also encountered frequently in remote acute stroke pathways, including among patients who receive thrombolytic treatment. These observations are well documented, but they should not be interpreted as direct evidence that telestroke lowers the threshold for treatment.

This review proposes threshold shift as a testable hypothesis rather than an established effect of telestroke. Remote assessment occurs within a distinctive clinical environment in which treatment is time sensitive, diagnostic information may remain incomplete, and decisions are shared between hub and spoke teams. Risk asymmetry, information constraints, and distributed responsibility may therefore influence how uncertainty is managed. However, current observational evidence cannot determine whether clinicians actually require less diagnostic certainty before recommending treatment, and alternative access-, workflow-, and case-mix explanations remain plausible.

The central challenge is therefore not to eliminate treatment under uncertainty. Some uncertainty is unavoidable when therapeutic benefit declines with time, and a final diagnosis of stroke mimic does not necessarily mean that the original treatment decision was inappropriate. A more meaningful objective is to improve the calibration of treatment decisions: preserving rapid treatment for patients with true ischemic stroke while allowing additional diagnostic clarification when this can be achieved without clinically important delay.

Future research should move beyond treatment rates as a surrogate for decision behavior. Prospective studies should measure clinicians’ diagnostic confidence, estimated probability of ischemic stroke, competing diagnoses, information available at the time of decision, and reasons for recommending or withholding thrombolysis. Comparing these variables across telestroke and bedside care would allow the threshold-shift hypothesis to be tested directly. Such evidence could help telestroke evolve from a system that primarily expands access to reperfusion therapy into one that also supports more transparent, measurable, and consistently calibrated decision-making under diagnostic uncertainty.
